# Cervical Interspinous Ligament Sprain in a 6-Year-Old Boy

**DOI:** 10.5334/jbsr.2873

**Published:** 2022-10-04

**Authors:** Thomas Saliba, Hanna Salame, Denis Tack

**Affiliations:** 1 Epicura Hospital, BE

**Keywords:** cervical spine injury, soft tissue injury, ligament injury, paediatric cervical spine, spine trauma

## Abstract

Paediatric cervical spine trauma, though rare, is difficult to detect as the injuries are often soft-tissue injuries and thus not visible using conventional radiography. A 6-and-a-half-year-old child presented with neck pain following a fall. A thorough radiological workup over several days demonstrated soft-tissue injuries, undetected by initial cervical X-rays, requiring MRI to definitively prove. The patient recovered with conservative treatment.

**Teaching Point:** Paediatric cervical spine injuries often present with soft tissue injuries, which can missed on X-rays and require further imaging to detect.

## Introduction

Paediatric cervical spine trauma is rare, with an incidence only half of that of the adult population (1–1.3%), generally due to motor vehicle crashes (MVCs) (57.51%), followed by falls and sports [1,2]. Paediatric patients tend to suffer far more serious sequelae, up to 60% suffering permanent neurological damage and 40 to 50% dying, with death being inversely proportional to age [2].

Importantly, children are more susceptible to spinal cord injury without radiographic abnormality than adults, though by age 8 to 10 the injury pattern resembles that of the adult population as the spine has matured [2].

## Case History

**Figure 1:**
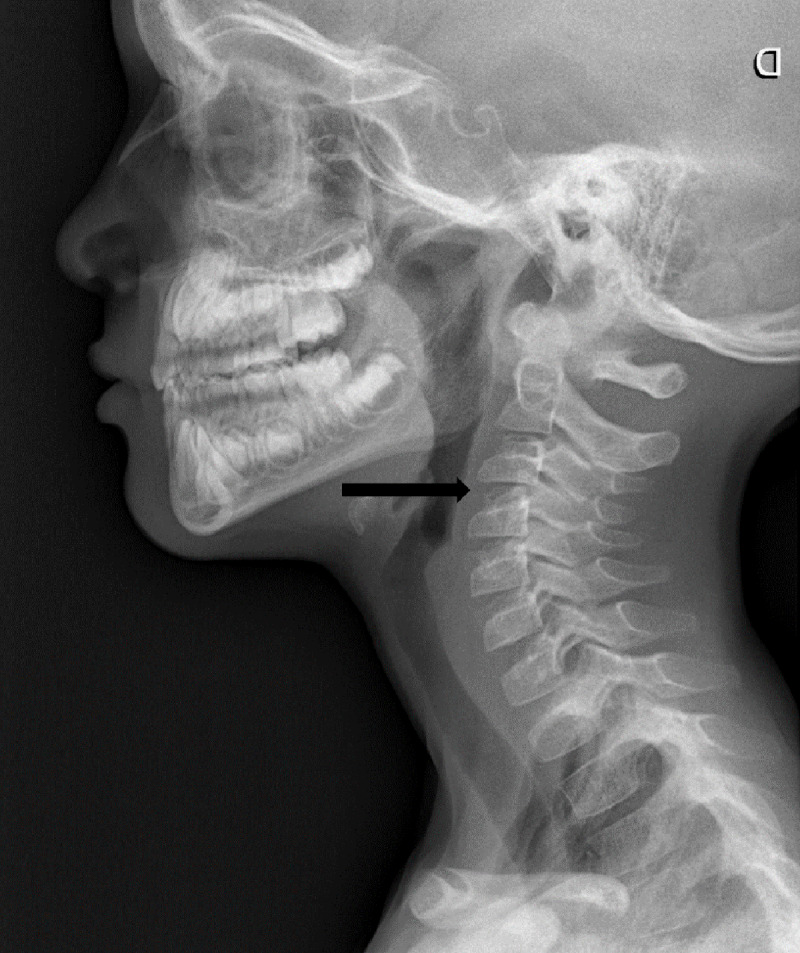
Initial lateral cervical X-ray where a discrete C3-C4 dislocation was described.

**Figure 2:**
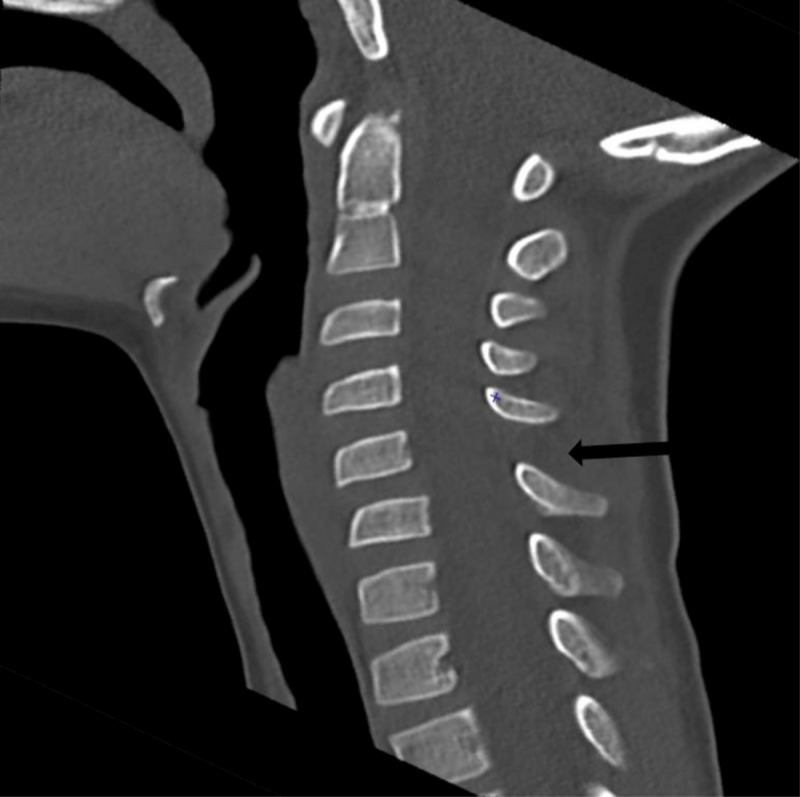
Follow-up CT exam revealing C4-C5 anterolisthesis and an increase in the corresponding interspinous space.

**Figure 3:**
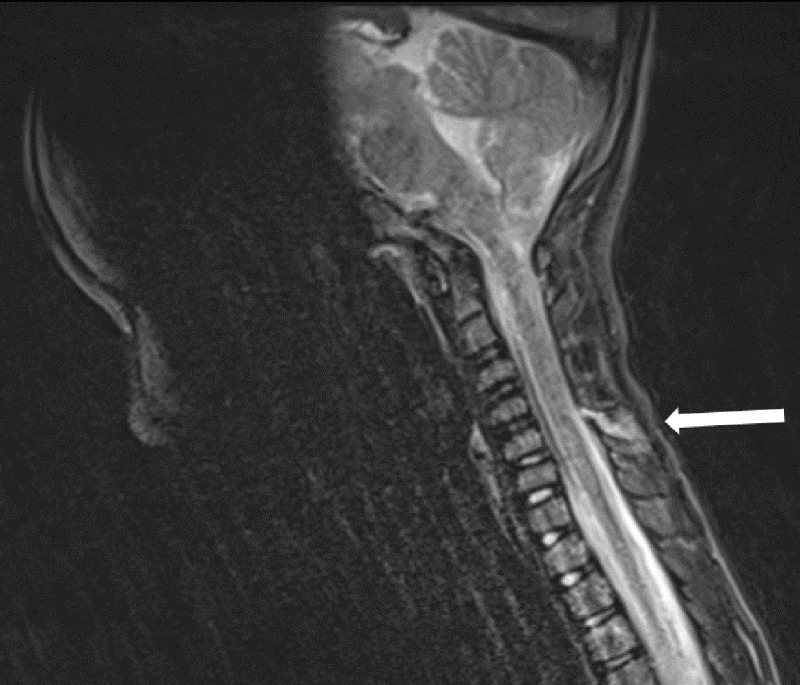
MRI showing C5-C6 interspinous ligament oedema.

**Figure 4:**
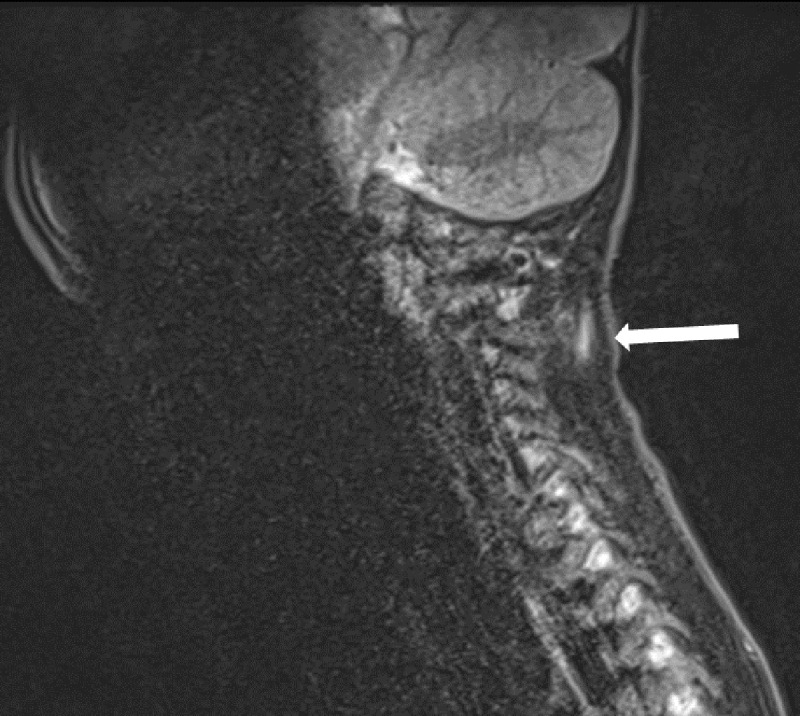
MRI showing left superficial posterior paramedian muscle oedema around C2.

A 6-and-a-half-year-old boy presented to the emergency department with neck pain after falling from a bouncy castle. Initially, a cervical X-ray was ordered where a C3-C4 retrolisthesis was described (Figure 1). The patient was discharged with a foam collar, ibuprofen, and a follow-up orthopaedic appointment and CT exam. The CT exam was performed three days later, revealing a 2mm C4-C5 anterolisthesis, an increased interspinous process space, but no fractures (Figure 2), therefore the previous X-ray’s findings weren’t confirmed. The following day an MRI revealed C5-C6 interspinous ligament oedema, suggestive of a sprain, and superficial posterior paraspinal muscle oedema around C2 (Figures 3, 4). An orthopaedic surgeon prescribed physiotherapy and saw the patient once more a month later, at which point the patient showed no signs of lasting damage.

## Comment

Most paediatric vertebral injuries involve the cervical spine, as opposed to lumbar and thoracic spine injuries in adults [2]. They also more commonly involve ligament injury instead of bone injury [2]. The reason is that children’s ligaments are more lax than in adults and disproportionately weaker, which – alongside a proportionally larger head, horizontal facets, anteriorly wedge-shaped vertebral bodies, and incomplete ossification – increases the freedom of motion in children’s cervical spines as well as making those under 8 more susceptible to spinal injuries [1,2].

Eight predictors of cervical spine injuries, the PERCARN (Pediatric Emergency Care Applied Research Network) criteria, allowing detection of up to 98% of cervical spine injuries, are altered mental status, focal neurological deficit, torticollis, substantial torso injury, neck pain, predisposing medical conditions, diving, and high-risk MVCs [2,3].

The Easter Association for the Surgery of Trauma (EAST) deems CT the best initial modality regardless, in contrast to the American College of Radiology’s recommendations, which prefer X-rays [3]. For high-risk patients, CT exam is the preferred initial modality [2,3].

Lateral radiography is the most sensitive view for paediatric spine injury, though only 74–85% sensitive for fractures [2,3]. The odontoid view is controversial, being difficult to obtain, and adding little diagnostic information according to some, but increasing sensitivity up to 94% according to others [2,3]. Flexion and extension radiographs are not recommended initially but may be useful to follow up suspected ligamentous laxity [2,3]. Evaluation of spinal alignment involves assessment of the craniocervical junction, the anterior vertebral and posterior vertebral lines, spinolaminar lines, and posterior spinous lines [2,3].

The use of CT as a screening tool is debated, with some studies showing superior sensitivity whilst others finding no differences for significant injuries when compared to standard radiographs [2]. It should be noted, however, that even a negative CT exam cannot fully exclude spinal injury without corroborating clinical findings, in which case an MRI may be needed if one is unable to fully clear a cervical spine within three days [2,3].

Finally, MRI is the most specific and sensitive method for paediatric spine trauma but has limited availability and is subject to other technical barriers like the need for anaesthesia [2]. However, MRI is the preferred method in acute neurological abnormalities [2].

In the paediatric population soft tissue and ligamentous injuries are more common and may be the only signs of injury [2,3]. These can present as soft tissue swelling, as was the case for our patient, or osseous misalignment [2]. Some signs, such as prevertebral oedema, epidural hematoma and interspinal or paraspinal oedema are present in over 50% of cervical spine injury cases [3].

Of the types of injuries than can occur AOSpine B injuries (distraction) occur most frequently in children under 3 and decrease with age. These tend to be injuries of the posterior tension band, seen as widening of the interspinous distance or uncovering of the facets [3].

The clinical significance of findings such as soft tissue contusion, oedema in the interspinous ligaments with intact anterior and middle pillars and isolated disruption of the nuchal ligament is unclear [3]. When a paediatric cervical spine lesion is detected, however, around 30–40% of will require surgery [3].

## Conclusion

Paediatric spine injuries are relatively rare, can be difficult to detect, and are an important entity due to their high morbidity, mortality, and the frequent necessity of surgery [2,3]. The imaging approach is both case and centre specific, with either radiography or CT being the preferred initial modality.
